# Factors Associated with Gastrointestinal Parasitic Infections among Young Population in Northeast Brazil

**DOI:** 10.1155/2016/6239434

**Published:** 2016-07-26

**Authors:** Juliana Vasconcelos Lyra da Silva, Gilberto Fontes, Célia Dias dos Santos, Rafael Vital dos Santos, Eliana Maria Mauricio da Rocha

**Affiliations:** ^1^Instituto de Ciências Biológicas e da Saúde da Universidade Federal de Alagoas, 57072-970 Maceió, AL, Brazil; ^2^Faculdade de Nutrição da Universidade Federal de Alagoas, 57072-970 Maceió, AL, Brazil; ^3^Universidade Federal de São João del Rei, Campus Centro Oeste, 35501-296 Divinópolis, MG, Brazil

## Abstract

*Background*. Intestinal parasitic infections constitute a major public health problem that is frequently associated with poverty, inadequate sanitation, and the nutritional status of the population.* Objective*. The aim of the present study is to investigate the possible association of parasitic infections, sanitary conditions, hygiene practices, and the nutritional and socioeconomic status of a poor youth population.* Methods*. A cross-sectional study was conducted with 367 children and adolescents inhabiting a substandard settlement in the urban area of Maceió (Alagoas State, Brazil). Data collection included socioeconomic status, anthropometric measurements, fecal sample examinations, and laboratory blood analysis. The identification of factors associated with gastrointestinal parasitic infections was undertaken through bi- and multivariate analyses.* Results*. Stool sample analysis obtained from 300 individuals revealed that 204 (68%) were infected with at least one parasite species and of these 130 (63.7%) were polyparasitized. No significant associations were identified between low height for age (stunted), parasitic infections, and polyparasitism. There was also no association between family income and parasitosis. However, low socioeconomic status proved to be a potential risk factor for parasitic infections.* Conclusion*. Actions must be taken to improve sanitation, housing, and environmental conditions in order to eliminate the risk factors for parasitic infections, and thereby guarantee a better quality of life for this population.

## 1. Introduction

Parasitic intestinal infections are more prevalent in school-aged children and cause a wide range of complications, including bowel obstruction, anorexia, anemia, diarrhea, and malabsorption [1]. Parasitic infections, undernutrition, and iron deficiency anemia (IDA) are common problems in developing countries and they are mainly due to poverty which contributes to food insecurity, unclean surroundings, and limited access to health care [2]. These diseases result in growth retardation and the impairment of neuropsychomotor development, immune function, productive ability, and intellectual capacity [1, 3].

Undernutrition and IDA are commonly associated with intestinal parasitosis, although some studies have raised doubts regarding such associations. Tsuyuoka et al. [4] confirmed the association of intestinal parasitic infections with malnutrition but not with anemia. However, Monteiro [5] considered intestinal helminthiasis and inadequate food intake to be prime factors in the pathophysiology of anemia and malnutrition. Muniz-Junqueira and Queiróz [6] found an association between only malnutrition and giardiasis, whereas Castro et al. [7] reported a correlation of anemia with* Entamoeba histolytica* infection. To date, the relationship of intestinal parasitic infections with some nutritional indicators remains not entirely clear. Therefore, the aim of this study is to investigate the associations that may exist between enteroparasitosis and nutritional and socioeconomic variables.

## 2. Materials and Methods

### 2.1. Study Design

**Figure 1:**
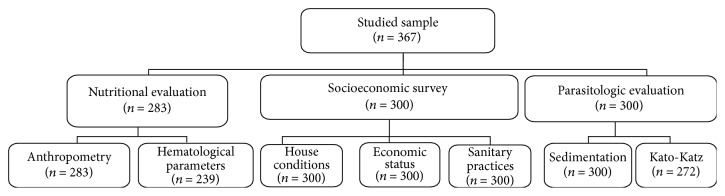
Flow chart of the number of participants included in each evaluation of the study.

A cross-sectional study was undertaken enrolling 367 children and adolescents, including 186 boys (50.7%) and 181 girls (49.3%) aged 0 to 15 years (mean 7.7 ± 4.2 years). The sample size was calculated based on an expected prevalence of 53%, an acceptable error of 10%, and a 99% confidence interval. This prevalence was obtained from a preliminary study conducted in the area [8]. This calculation returned a minimal sample size of 273 children, which was increased to compensate for anticipated losses during follow-up. A flow chart indicating the number of participants for each evaluation is presented in Figure 1.

### 2.2. Study Area

The subjects lived in the Reginaldo Valley, a substandard settlement in the urban area of Maceió (Alagoas State, Brazil), which covers approximately 60,000 m^2^ with an estimated population of 65,000 people. The region was endemic for lymphatic filariasis until 2005 [9]. Despite being an ideal setting for the transmission of enteroparasites, the epidemiological situation of this area has not been described. The households in this study were randomly selected.

### 2.3. Socioeconomic Survey

A household survey was undertaken to collect the socioeconomic data. The variables were divided into three groups: living conditions, economic indicators, and behavior related to sanitary education. The socioeconomic level of the participants was classified according to the Brazilian Economic Classification Criterion ABEP-2008 [10]. Social categorization from class A to class E (from highest to lowest income class) is based mainly on minimum wage per month per family, the parental educational level, and possession of household items (TV, stove, refrigerator, etc.).  Table 1 shows the characteristics of the studied population.

### 2.4. Parasitological Analysis

Fecal samples were processed and evaluated using techniques based on spontaneous sedimentation [11] and Katz et al. [12] techniques. To be regarded as infected, the patient had to present a positive diagnosis for an intestinal parasite in at least one of the tests performed.

### 2.5. Anthropometric Evaluation

Anthropometric evaluations were performed by trained personnel, following the technical procedures of the Brazilian Ministry of Health [13]. Height for age (H/A) and body mass index for age (BMI/A) were calculated and expressed as *Z* scores [14, 15]. The data was statistically analyzed using WHO AnthroPlus [16] and Microsoft Office Excel® software.

### 2.6. Analysis of Hemoglobin, Ferritin, and Iron

Iron status was determined by measuring the hemoglobin (Hb), ferritin (SF), and iron (SI) levels in blood collected from each participant through venipuncture. The cyanmethemoglobin method, an automated chemiluminescence system (ACS 180®, Bayer HealthCare), and spectrophotometry were used to determine, respectively, Hb, SF, and SI levels [17, 18], Table 2.

### 2.7. Statistical Analysis

Bivariate (chi-square) and multivariate (logistic regression) statistical analyses were performed to evaluate the association between variables. Odds ratios (OR) (95% CIs) were calculated as a measurement of the strengths of associations. Relevant variables that achieved *p* value < 0.20 in the bivariate analyses were included in the stepwise logistic regression model. For the multivariate logistic regression final model only variables associated with the dependent variable (intestinal parasitic infection) were retained (*p* < 0.05). Variables with more than two categories were redefined into* dummy* variables (categories 0-1). Statistical analysis was done using the Statistical Package for Social Sciences (SPSS), version 11.5, and STATA Statistical Software, version 10.

### 2.8. Ethical Considerations

The study was approved by the Research Ethics Committee of the Federal University of Alagoas (number 006491/2004-85). Only subjects whose parent/legal guardian signed a consent form participated in this study.

## 3. Results and Discussion 

Overall, out of the 300 individuals examined for intestinal parasites, 204 (68%) were infected with at least one species. High rates of prevalence have already been found in Brazil in areas with precarious socioeconomic and hygienic conditions. In studies conducted by Gomes et al. [19] in the state of Rio de Janeiro and by Buschini et al. [20] in the city of Guarapuava, Paraná State, parasitic infections were diagnosed in 63.2% of homeless people and in 75.3% of school-aged children, respectively. Fontes et al. [21] found that 92% of school-aged children, ranging from 5 to 18 years old, living in the city of Barra de Santo Antônio in the state of Alagoas, were infected with at least one species of intestinal parasite.

**Figure 2:**
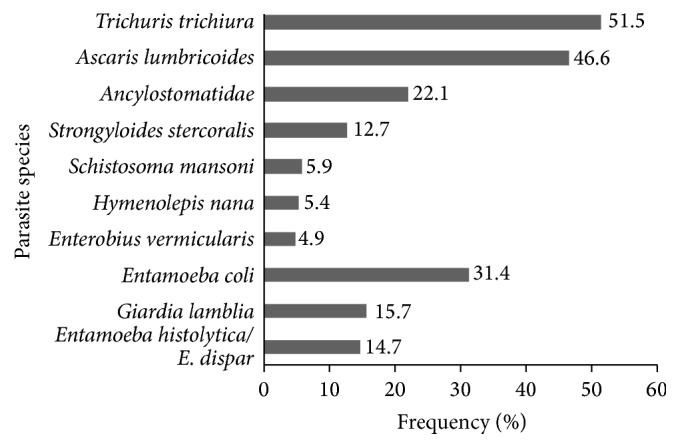
Frequency of parasite species identified in stool samples from children aged 0 to 15 years, living in a substandard settlement in Maceió, Alagoas.

Eleven species of parasites were detected in this study, and 130 (63.7%) of the infected individuals suffered from polyparasitism. The number of parasite species harbored per host ranged from one to six, with a mean of 2.1 (±1.1) and median of two parasite species per host. Helminthic infections were more common than protozoal infections, mainly* Trichuris trichiura* (51.5%) and* Ascaris lumbricoides* (46.6%) (Figure 2). Ascariasis was also the most frequently recorded parasitic infection in school-aged children from Alagoas [3, 19]. Similar results were found by Lander [22] in a study conducted in the northeast region of Brazil. In several Latin American communities, infection with* A. lumbricoides* often affects more than 20% [23] of the population.

In bivariate analysis, the variables that had significant positive associations (95% CI) with intestinal parasite infections were socioeconomic status, footwear use, and number of household members (Table 3).

In the complete multivariate model, in addition to the aforementioned variables, indoor toilet, family income, and contact with natural water sources were also included as variables. The multivariate analysis is shown in Table 4. Only socioeconomic status (class E) and households with more than five individuals maintained significant associations with 95% CIs.

Nematian et al. [24] studied school-aged children in Tehran (Iran) and demonstrated a relationship between number of siblings and parasitic infection. It is likely that having a large number of people in a household results in less attention to positive health habits. In addition, larger families usually have a lower socioeconomic status.

Results from different investigations have been in disagreement with regard to both stunting and undernutrition and the relationship these have with parasitic infection. No such relationship was found to exist in the present study. Similar results were obtained in the investigation conducted by Casapía et al. [25]; these authors studied preschool-aged children and found no evidence of a relationship between underweight and parasitic infections or polyparasitism. In contrast, Alvarado and Vásquez [26] reported a higher prevalence of acute malnutrition in children with polyparasitism and trichuriasis, while Phathammavong et al. [27] described a greater prevalence of underweight and nutritional stunting among school-aged children infected with intestinal parasites.

According to Santos [3], the lack of a correlation between nutritional deficits and parasitic infections could be explained mainly by the decline in the occurrence of malnutrition over the last few decades in Brazil as well as by the decrease of inadequate diets, by low levels of infection, and by the indiscriminate use of antiparasitic drugs obtained without a medical prescription.

A survey conducted in the semiarid region of Alagoas State revealed that 87.3% of the families were classified in the lowest economic stratum [28]. Furthermore, the state had the worst child development index score in Brazil (CDI = 0.473) [29]. Therefore, establishing a plan of action targeted mainly against soil-transmitted parasites requires urgent attention. Social scientists in Brazil have repeatedly addressed the need to prioritize investment in sanitation and make sanitation one of the most important public policy initiatives [30]. The lives of these children are hampered by the same widespread poverty documented in other studies. Access to basic health services, education, and adequate housing will not become a reality unless specific actions are taken to improve the quality of life among people living in unsanitary conditions.

## Figures and Tables

**Table 1:** Demographics and behavioral characteristics of children aged 0 to 15 years, living in a substandard settlement in Maceió, Alagoas, Brazil.

Variables	Number of participants (%)
Gender	
Male	186 (50.7)
Female	181 (49.3)

Age group	
0–5	133 (36.2)
6–10	135 (36.8)
11–15	99 (27.0)

Number of household members	
≤5	157 (52.3)
>5	143 (47.7)

Toilet type in use	
No toilet	43 (14.4)
Indoor toilet with flush	174 (58.0)
Indoor toilet without flush	22 (7.3)
Pit latrine with water	37 (12.3)
Pit latrine	24 (8.0)

Social status^*∗*^	
C1	2 (0.7)
C2	21 (7.0)
D	141 (47.0)
E	136 (45.3)

Family income (MW^*∗∗*^/family/month)	
Unknown	44 (14.7)
<1	97 (32.3)
1	91 (30.3)
1-2	50 (16.7)
2-3	16 (5.3)
3–5	2 (0.7)

Footwear use	
Yes	123 (41.0)
No	177 (59.0)

Contact with natural water sources	
Yes	88 (29.3)
No	212 (70.7)

^*∗*^Economic classification proposed by ABEP [10]; ^*∗∗*^MW: minimum wage.

**Table 2:** Cut-off point criteria for nutritional status and hematological parameters according to age, for the evaluation of a sample of people aged 0 to 15 years in Maceió, Alagoas, Brazil.

Anthropometric indices and hematological parameters						Reference
Nutritional status (H/A)						
Classification	Very low height for age	Low height for age	Adequate height for age			[14, 15]
Score	*Z* < −3	−3 ≤ *Z* < −2	*Z* ≥ −1			

Nutritional status (BMI/A)						
Classification	Malnourished	Risk	Eutrophy	Overweight	Obese	[14, 15]
Score	*Z* <–2	−2 ≤ *Z* < −1	−1 ≤ *Z* ≤ 1	1 < *Z* ≤ 2	*Z* > 2	

Hemoglobin						
Age	6–59 months	5–11 years	12–15 years	≥15 years		[17]
Cut-off (g/dL)	<11.0	<11.5	<12.0	13.0		

Ferritin						
Age	<5 years	≥6 years				[17]
Cut-off (ng/mL)	≥12	≥15				

Serum iron						
Age	Age independent					[18]
Cut-off (*μ*g/mL)	<50					

**Table 3:** Bivariate analysis for factors potentially associated with intestinal parasitic infection among children aged 0 to 15 years, from a substandard settlement in Maceió, Alagoas, Brazil.

Variables	Number of individuals	Infected		Odds ratio	95% CI	*p* value
		*n*	%			
Age group						
0–5	63	42	66.7	1		
6–10	77	49	63.6	0.9	0.4–1.8	0.71
11–15	41	30	73.2	1.4	0.6–3.3	0.48

Gender^*∗*^						
Male	92	59	64.1			
Female	89	62	69.7	1.3	0.7–2.4	0.43

Social status^*∗∗∗*^						
C and D	105	63	60.0			
E	76	58	76.3	2.1	1.1–4.1	0.02^*∗∗*^

Family income^*∗∗∗∗*^						
>1 minimum wage	46	27	58.7			
≤1 minimum wage	103	71	68.9	1.6	0.8–3.2	0.22

Number of household members^*∗∗∗*^						
≤5	100	59	59.0			
>5	81	62	76.5	2.3	1.2–4.3	0.01^*∗∗*^

Indoor toilet						
Yes	120	78	65.0			
No	61	43	70.5	1.3	0.7–2.5	0.46

Footwear use^*∗∗∗*^						
Yes	78	47	60.3			
No	103	74	71.8	1.7	0.9–3.14	0.10^*∗∗*^

Contact with natural water sources						
No	135	88	65.2			
Yes	46	33	71.7	1.4	0.7–2.8	0.41

Anemia						
No	161	109	67.7			
Yes	20	12	60.0	0.7	0.3–1.9	0.49

Low level of ferritin						
No	152	100	65.8			
Yes	29	21	72.4	1.4	0.6–3.3	0.49

Low level of iron						
No	117	78	66.7			
Yes	64	43	67.2	1.0	0.5–2.0	0.94

Growth stunting						
No	160	107	66.9			
Yes	21	14	66.7	1.0	0.4–2.6	0.98

Low weight						
No	173	114	65.9			
Yes	8	7	87.5	3.6	0.4–30.1	0.23

^*∗*^Control variables selected for multivariate logistic regression model.

^*∗∗*^Statistically significant difference.

^*∗∗∗*^Selected variables for multivariate logistic regression (*p* < 0.20).

^*∗∗∗∗*^32 participants without income information.

**Table 4:** Final model multivariate logistic regression analysis for factors potentially associated with intestinal parasite infection, among children aged 0 to 15 years, from a substandard settlement in Maceió, Alagoas, Brazil.

Risk variables	Adjusted odds ratio	95% CI	*p* value
Social status, E^*∗∗*^	2.4	1.2–4.6	0.01^*∗*^
>5 household members^*∗∗∗*^	2.5	1.3–4.8	0.01^*∗*^

^*∗*^Statistical significance.

^*∗∗*^Reference categories: C and D.

^*∗∗∗*^Reference category: ≤ 5 household members.
